# Theory of Mind in migraine and medication-overuse headache: A cross-sectional study

**DOI:** 10.3389/fneur.2022.968111

**Published:** 2022-08-26

**Authors:** Marina Romozzi, Sonia Di Tella, Eleonora Rollo, Paolo Quintieri, Maria Caterina Silveri, Catello Vollono, Paolo Calabresi

**Affiliations:** ^1^Dipartimento Universitario di Neuroscienze, Università Cattolica del Sacro Cuore, Rome, Italy; ^2^Neurologia, Dipartimento di Scienze dell'invecchiamento, Neurologiche, Ortopediche e della Testa-Collo, Fondazione Policlinico Universitario Agostino Gemelli IRCCS, Rome, Italy; ^3^Dipartimento di Psicologia, Università Cattolica del Sacro Cuore, Milan, Italy; ^4^Neurofisiopatologia, Dipartimento di Scienze dell'invecchiamento, Neurologiche, Ortopediche e della Testa-Collo, Fondazione Policlinico Universitario Agostino Gemelli IRCCS, Rome, Italy

**Keywords:** Theory of Mind, medication-overuse headache, migraine, alexithymia, ToM abilities, Reading the Mind in Eyes Test, depression, anxiety

## Abstract

**Background:**

Theory of Mind (ToM) is the ability to predict and anticipate others' behaviors through the mental state attribution process. This study aims to investigate the ToM in patients with medication-overuse headache (MOH) and episodic migraine (EM) and to compare it with healthy controls (HC).

**Methods:**

This study enrolled patients with MOH, patients with EM, and HC. ToM was assessed through the Theory of Mind Assessment Scale (ThOMAS), which includes four subscales: Scale A, I-Me, Scale B, Other-Self, Scale C, I-Other, and Scale D, Other-Me, through the Reading the Mind in the Eyes test (RMET), which measures complex emotion recognition, and through the Toronto Alexithymia Scale (TAS-20), which measures alexithymia. Concomitant psychiatric disturbances were evaluated through the Hamilton Anxiety Rating Scale, the Hamilton Depression Rating Scale, and the Dissociative Experiences Scale-II.

**Results:**

The study involved 21 patients with EM, 22 patients with MOH, and 18 HC. In all the four subscales of the ThOMAS, there was a significant difference between HC, EM, and MOH patients: Scale A (*p* = 0.009), Scale B (*p* = 0.004), Scale C (*p* = 0.039), and Scale D (*p* = 0.008). In the RMET, MOH patients had worse performances than EM patients and HC (*p* = 0.039). MOH group exhibited higher levels of alexithymia when compared to the HC (*p* = 0.033) and higher levels of anxiety than HC (*p* = 0.001).

**Conclusion:**

MOH patients showed a subtle psychopathological pattern characterized by impaired social adaptation.

## Introduction

Migraine is one of the most common debilitating diseases affecting more than 1 billion people worldwide. Medication-overuse headache (MOH) is a chronic headache that occurs in patients with established primary headache disorders who overuse different acute medications to relieve the symptoms (1, 2). Migraine is frequently comorbid with psychiatric conditions such as bipolar and anxiety disorders and major depression, negatively impacting the quality of life (QoL) and disability (3).

Psychiatric disorders seem to be more prevalent in MOH than in patients with migraine (1, 4). Indeed, patients with MOH may have higher rates of obsessive-compulsive disorder (OCD), and they may share common aspects with other forms of drug-addition and common neural networks (5, 6). Moreover, psychiatric comorbidities in patients with migraine represent an important factor in determining the transformation from episodic to chronic headache (7).

Social cognition is a neurocognitive capacity underlying social interaction, which allows us to perceive, process, and interpret social information. It has a multicomponent nature that involves empathy, emotional/social perception, and Theory of Mind (ToM). ToM is the ability to predict and anticipate others' behaviors through mental state attribution process (8).

Recent works have identified two different components by considering ToM as a multifaced psychological construct. Specifically, the affective component is responsible for understanding others' emotions, while the cognitive one is related to the knowledge of others' mental states, beliefs, thoughts, and intentions (8).

Alexithymia is characterized by difficulties in identifying, differentiating and describing feelings, and some authors hypothesized that alexithymia could be related to ToM and, in particular, to the affective component (9). Besides, previous studies suggested that alexithymia could be a psychological trait of patients with migraine (10).

Furthermore, previous literature supports the existence of an association between social cognition disorders such as ToM deficits and alexithymia and dissociative symptoms (11). People who suffer from severe dissociative experiences might have difficulties in metalizing and regulating emotions with a negative view of the self.

Although few studies have been conducted in children with migraine, showing the presence of social cognition impairment (12), studies on ToM in different forms of migraine in the adult population are currently underreported.

Bouteloup et al. conducted a study on 23 patients with a diagnosis of migraine, demonstrating that these patients present difficulties in inferring mental states compared to HCs. However, the study included a small cohort of patients with both episodic migraine and chronic migraine with and without medication overuse (13). To the best of our knowledge, studies on ToM and MOH have never been conducted.

Because of the intrinsic importance of social functioning in QoL and the mediating effect of social cognition on social functioning, ToM could be also related to the quality of life (14).

The main aims of this study are to assess the Theory of Mind (ToM) and behavioral profile in a cohort of adult patients with MOH, episodic migraine (EM), and healthy volunteers.

## Materials and methods

### Setting and participants

This cross-sectional observational study was conducted in a Headache Tertiary Center (Fondazione Policlinico Universitario A. Gemelli IRCCS in Rome, Italy) from November 2020 to November 2021.

We enrolled patients with a diagnosis of MOH, patients with a diagnosis of EM, and age-matched healthy controls (HC) without a history of headache. Inclusion criteria were age >18 years and informed consent to participate. Patients with migraine and MOH groups fulfilled the International Classification of Headache Disorders, 3rd edition (ICHD-3) criteria for the diagnosis of EM and MOH (2). HC reported no previous diagnosis of headache disorders and did not fulfill the criteria for primary headache disorders after a detailed interview performed by four neurologist headache specialists. We excluded patients with a diagnosis of a secondary headache, subjects who refused to give informed consent, patients with cognitive decline or mental illness, and non-Italian native speakers.

Episodic migraine is characterized by 14 or fewer days of headache per month; MOH was defined as a chronic headache that occurs >15 days/month in patients who regularly overuse headache medications (more than 10 or 15, depending on the medication) for more than 3 months (2). The study conforms to the ethical guidelines of the 1975 Declaration of Helsinki, as reflected in a priori approval by the institution's human research committee at each participating study site. The study was approved by the Ethic Committee of Fondazione Policlinico A. Gemelli IRCCS.

### Study aims

The main aims of the study were (1) to assess ToM abilities and (2) to characterize the behavioral profile of a cohort of adult patients with MOH, comparing them to a group of patients with episodic migraine (EM) and healthy volunteers. In more detail, to achieve the first goal, we administered the Theory of Mind Assessment Scale (15), a useful tool that provides a complete and detailed evaluation of ToM abilities. We hypothesized that ToM abilities of MOH groups were different compared to HC and EM groups, but we also wanted to investigate in detail whether specific components or sub-skills were more impaired than others. We hypothesized to find a difficulty in the comprehension of own mental states in MOH group. To achieve the second aim, we also administered psychobehavioral questionnaires evaluating theoretical constructs related to ToM including affective symptoms (anxiety and depression), alexithymia, and dissociation.

### Cognitive screening

All subjects were screened for cognitive impairment with the Montreal Cognitive Assessment (MoCA) (16), which explores several cognitive domains: memory, attention, and language and orientation, visuospatial, and executive functions domains. The MoCA total score ranges from 0 to 30 with higher scores indicating better performances. After adjusting for age and education, the cutoff for cognitive impairment is 17.54 according to Italian normative data. The exact formula for the adjusted MoCA was “raw MoCA score – 4.228 9 [log10(100 – age) – 1.58] – 3.201 9 [square root (years of education) – 3.25]” (17).

### Assessment of migraine features

Migraine features were collected from a neurologist and headache specialist. The characteristics of migraine and accompanying symptoms were clarified through a structured interview.

We collected information about sociodemographic data, general medical history, age at onset of headache, duration of headache attacks, frequency of headaches (monthly headache days, MHD), pain characteristics, accompanying symptoms, aura symptoms, history of acute and preventive medication use, and number of drugs per month.

We assessed the severity and the headache-related disability and the headache's impact on work through different scales: the Headache Impact Test-6 (HIT-6) and the Migraine Disability Assessment Scale (MIDAS).

### Assessment of Theory of Mind

ToM was assessed using the Reading the Mind in the Eyes test (RMET), the Theory of Mind Assessment Scale (ThOMAS), and the Toronto Alexithymia Scale (TAS-20).

The RMET evaluates affective ToM. Stimuli consist of photographs of actors' eyes expressing a certain emotion. The test consists in choosing the right mental state among four choices (e.g., puzzled, nervous, insisting, or contemplative) that best defines what the person in each photograph thinks or feels. Accuracy on this task is quantified by how many of the target adjectives the participant correctly selects for the 36 photographs displayed. Total score ranges from 0 to 36 (18).

The ThOMAS is a semistructured interview (15). It is a direct investigation because it is the subject himself who expresses his knowledge about one's own and other person's mental states. Stimulated by appropriate questions about what thoughts, emotions, beliefs, etc. and what are their causes and reciprocal relations, the subject reflects and theorizes about her/his concept of mind. ThOMAS is composed of open-ended questions, which leave the interviewee free to express and articulate her/his thoughts. The interviewer, when this is not already done by the interviewee, systematically asks for real examples to enrich and contextualize the answers. The interview questions are organized into four scales, reflecting the different domains of knowledge in which ToM can manifest itself:

- Scale A, I-Me. The knowledge that the interviewee has of his/her mental states (e.g., “I am unhappy”).

- Scale B, Other-Self. The knowledge that other people have of their mental states (e.g., “Other people think they are successful”).

- Scale C, I-Other. The knowledge that the subject assumes other people have of his/her mental states (e.g., “Other people think I am inept”).

- Scale D, Other-Me. The subject's knowledge of other people's mental states (e.g., “I believe that other people get what they want”).

Each scale comprises three subscales that analyze the dimensions of Awareness, Relation, and Realization of mental states.

Awareness explores the ability of the participant to recognize and discriminate beliefs, desires, and emotions of oneself and other people.

Relation investigates the participant's ability to understand causal relationships between different mental states and recognize the resulting behaviors.

Realization investigates the ability of the participant to adopt effective behaviors to achieve the desired objective.

We also calculated sub-scores of different types of dimensions: Desires, Beliefs, Positive emotions, and Negative emotions.

With the consent of the participants, all ThOMAS interviews were recorded and rated by two independent judges according to the rating instructions. Each judge assigned for each item a score ranging from 0 to 4. The two judges achieved a significant level of interreliability on their first decision. For the decision of the final score, the two judges discussed each item where they diverged until they reached a full agreement. In case they do not reach an agreement, a third judge was called in.

The TAS-20 (19) is a 20 multiple-choice self-report questionnaire developed to evaluate the three components of alexithymia: Difficulty Identifying Feelings (DIF), Difficulty Describing Feelings (DDF), and Externally Oriented Thinking (EOT). The cutoff for high alexithymia on the TAS-20 total score is >61 out of a total score of 100. Thus, scores ≤ 51 indicate the absence of alexithymia, scores ≥61 indicate the presence of alexithymia, and scores of 52–60 suggest possible alexithymia.

### Other psychopathological evaluations

Concomitant psychiatric conditions were evaluated through different scales: Hamilton Anxiety Rating Scale (HAM-A), Hamilton Depression Rating Scale (HAM-D), and The Dissociative Experiences Scale-II (DES-II).

The HAM-A (20) is a clinician-rated evaluation that assesses the severity of anxiety. The HAM-A score ranges are mild anxiety from 8 to 14; moderate from 15 to 23; severe ≥24 (scores ≤ 7 are considered to represent no/minimal anxiety).

The HAM-D (21) is used to evaluate the severity of depression. The HAM-D score ranges are mild depression from 10 to 13; moderate depression from 14 to 17; severe >17 (score ≤ 9 are considered to represent no/minimal depression).

The DES-II (22) is a 28-item self-report questionnaire that evaluates dissociative symptoms such as depersonalization, de-realization, absorption, and amnesia. Participants are asked to rate each item as the percentage of the time in which they experience these symptoms, using a Likert-type scale ranging from 0% (never) to 100%, (always). The mean of all item scores ranges from 0 to 100%, with a cutoff score of 30 for psychopathology. For our research, we used the Italian translation of the DES-II.

### Statistical analyses

Descriptive statistics were used to describe the demographic and clinical features of the sample. Numerical variables were described using the following measures: mean and standard deviation. Categorical variables were presented as absolute number (*n*) and percentage (%) and compared with the chi-squared χ^2^ test. The distribution of each numerical variable was checked with Shapiro–Wilk test, and parametric or non-parametric analyses were performed accordingly.

We performed non-parametric ANOVA (Kruskal–Wallis test) to compare the three groups (HC, EM, and MOH) on each variable. Finally, the performances in the ToM battery were correlated with the number of monthly headache days (MHD) and the monthly number of symptomatic drugs. Pearson's or Spearman's rho correlation coefficients were calculated according to the distribution of the variable. The statistical significance was set at *p* < 0.05. We applied Bonferroni's correction to adjust for multiple comparisons.

## Results

The study cohort involved 21 patients with EM (mean age of 37.81 ± 15.38 years) and 22 patients with MOH (mean age of 37.23 ± 15.51 years). The HC were 18 subjects with a mean age of 33.22 ± 12.75 years. The three groups did not differ in age (*p* = 0.720) and global cognitive functioning (MoCA: *p* = 0.174); all the subjects obtained MoCA scores above the cutoff of cognitive impairment (17.54).

The EM group comprised three males and 18 females, the MOH group comprised three males and 19 females, and the HC group comprised nine females and nine males.

The clinical and demographic characteristics, including migraine features of the overall cohort, are summarized in Table 1. The main results are summarized in Tables 2, 3.

**Table 1:** Sociodemographical description of the sample.

**Variable**	**HC**		**EM**		**MOH**		***p***	**Pairwise comparisons**		
								**HC vs EM**	**HC vs MOH**	**EM vs MOH**
								**pBonf**	**pBonf**	**pBonf**
Age (Mean, SD)	33.22	12.57	37.81	15.38	37.23	15.51	0.720 #	n.s.	n.s.	n.s.
Education (Mean, SD)	17.67	2.22	16.29	2.99	14.50	4.44	**0.003 #**	0.388	**0.002**	0.166
Montreal cognitive assessment test (MoCA) (Mean, SD)	25.37	2.80	23.43	3.68	23.22	2.88	0.174 #	n.s	n.s	n.s
Sex M/F (*n*, %)	9 (50.0%)/9(50.0%)		3 (14.3%)/18 (85.7%)		3(13.6%)/19 (86.4%)		**0.012** **°**	**0.016**	**0.013**	0.951
Monthly headache days (MHD) (Mean, SD)	N/A	N/A	6.86	3.29	22.73	6.33	**<0.001** §			
Monthly number of symptomatic drugs (Mean, SD)	N/A	N/A	5.29	2.97	28.55	30.63	**<0.001** §			
Headache impact test−6 (HIT_6)	N/A	N/A	60.48	6.65	63.32	9.99	**0.010** §			
Migraine disability assessment (MIDAS)	N/A	N/A	28.38	34.72	87.09	63.74	**<0.001** §			

HC, healthy controls; EM, episodic migraine; MOH, medication-overuse headache; MHD, monthly headache days; M, males, F, females; SD, standard deviation; n.s., not significant; N/A, not applicable; #, non-parametric Kruskal–Wallis ANOVA; Chi-squared X^2^ test; §, Mann–Whitney U-test. Statistical significant results (*p* < 0.05) are shown in bold type. Bold values represents statistically significant.

**Table 2:** Performances of HC, EM, and MOH groups in ToM tasks compared with nonparametric Kruskal–Wallis ANOVA.

**Variable**	**HC**		**EM**		**MOH**		***p***	**Pairwise comparisons**		
	**Mean**	**SD**	**Mean**	**SD**	**Mean**	**SD**		**HC vs EM**	**HC vs MOH**	**EM vs MOH**
								**pBonf**	**pBonf**	**pBonf**
RMET (0-36)	27.39	3.35	26.14	3.51	24.76	3.28	**0.039**	0.598	**0.033**	0.580
**ThOMAS**										
**Scales**										
Scale A, I-Me (1–4)	2.98	0.19	2.94	0.42	2.60	0.53	**0.009**	0.999	**0.039**	**0.019**
Scale B, Other-Self (1–4)	2.92	0.25	2.70	0.48	2.42	0.63	**0.004**	0.769	**0.004**	0.088
Scale C, I-Other (1–4)	2.94	0.14	2.79	0.30	2.58	0.50	**0.039**	0.637	**0.033**	0.525
Scale D, Other-Me (1–4)	2.99	0.25	2.76	0.34	2.59	0.46	**0.008**	0.202	**0.006**	0.569
**Dimensions**										
Awareness (1–4)	2.91	0.19	2.82	0.31	2.65	0.52	0.246	n.s.	n.s.	n.s.
Relation (1–4)	3.00	0.15	2.83	0.33	2.63	0.47	**0.017**	0.343	**0.013**	0.551
Realization of mental states (1–4)	2.98	0.28	2.75	0.44	2.32	0.60	**0.001**	0.630	**0.001**	**0.026**
**Sub-scores of different types of dimensions**										
Beliefs (1–4)	2.83	0.42	2.67	0.51	2.31	0.64	**0.015**	0.999	**0.018**	0.118
Desires (1–4)	2.95	0.16	2.80	0.37	2.57	0.54	**0.023**	0.690	**0.019**	0.331
Positive emotions (1–4)	3.01	0.17	2.84	0.29	2.56	0.52	**0.004**	0.406	**0.003**	0.183
Negative emotions (1–4)	3.02	0.22	2.84	0.31	2.62	0.47	**0.008**	0.305	**0.006**	0.373

HC, healthy controls; EM, episodic migraine; MOH, medication-overuse headache; RMET, Reading the Mind in the Eyes; ThOMAS, Theory of Mind Assessment Scale; n.s., not significant; SD, standard deviation; pBonf, p-value adjusted for Bonferroni's correction. Statistical significant results (*p* < 0.05) are shown in bold type. Bold values represents statistically significant.

**Table 3:** Results of psychopathological evaluations of HC, EM, and MOH groups compared with non-parametric Kruskal–Wallis ANOVA.

**Variable**	**HC**		**EM**		**MOH**		***p***	**Pairwise comparisons**		
	**Mean**	**SD**	**Mean**	**SD**	**Mean**	**SD**		**HC vs EM**	**HC vs MOH**	**EM vs MOH**
								**pBonf**	**pBonf**	**pBonf**
Hamilton Anxiety Rating Scale (HAM-A)	7.94	6.65	12.71	6.80	19.77	11.43	**0.001**	0.255	**0.001**	0.109
Hamilton Depression Rating Scale (HAM-D)	9.83	6.17	10.81	4.55	14.36	7.74	0.101	n.s.	n.s.	n.s.
Toronto Alexithymia Scale (TAS-20)	50.35	6.35	56.10	6.67	58.91	12.31	**0.023**	0.066	**0.033**	0.999
Difficulty Describing Feelings (DDF)	12.65	2.74	14.19	3.08	14.43	4.40	0.317	n.s.	n.s.	n.s.
Difficulty Identifying Feelings (DIF)	12.29	3.35	14.52	4.76	17.05	7.01	0.104	n.s.	n.s.	n.s.
Externally Oriented Thinking (EOT)	25.41	3.39	27.38	2.80	27.43	5.56	0.176	n.s.	n.s.	n.s.
Dissociative Experiences Scale-II	8.19	8.43	10.71	7.80	12.00	10.21	0.350	n.s.	n.s.	n.s.

HC, healthy controls; EM, episodic migraine; MOH, medication-overuse headache; n.s., not significant; SD, standard deviation; pBonf, p-value adjusted for Bonferroni's correction. Statistical significant results (*p* < 0.05) are shown in bold type. Bold values represents statistically significant.

### MOH group

The pre-existing primary headache subtype in MOH patients was migraine for nine patients (40.9%), tension-type headache (TTH) for two patients (9.1%), and mixed headaches for 11 patients (50%). The mean monthly headache days (MHD) was 22.73 ± 6.33. The mean monthly number of symptomatic drugs was 28.55 ± 30.63. A total of 20 patients (90.9%) received preventive treatment. The overused medications were analgesics (40.9%), triptans (36.4%), opioids (9.1 %), or combination drugs (13.6%). For the MOH group, the mean MIDAS score was 87.09 ± 63.74, and the mean HIT-6 score was 63.32 ± 9.99 (Table 1).

### EM group

For the EM group, the mean MHD was 6.86 ± 3.29. For the MOH group, the mean MIDAS score was 28.38 ± 34.72, and the mean HIT-6 score was 60.48 ± 6.65. The mean monthly number of symptomatic drugs was 5.29 ± 2.97 (Table 1).

### Theory of Mind Assessment Scale (ThOMAS)

**Figure 1:**
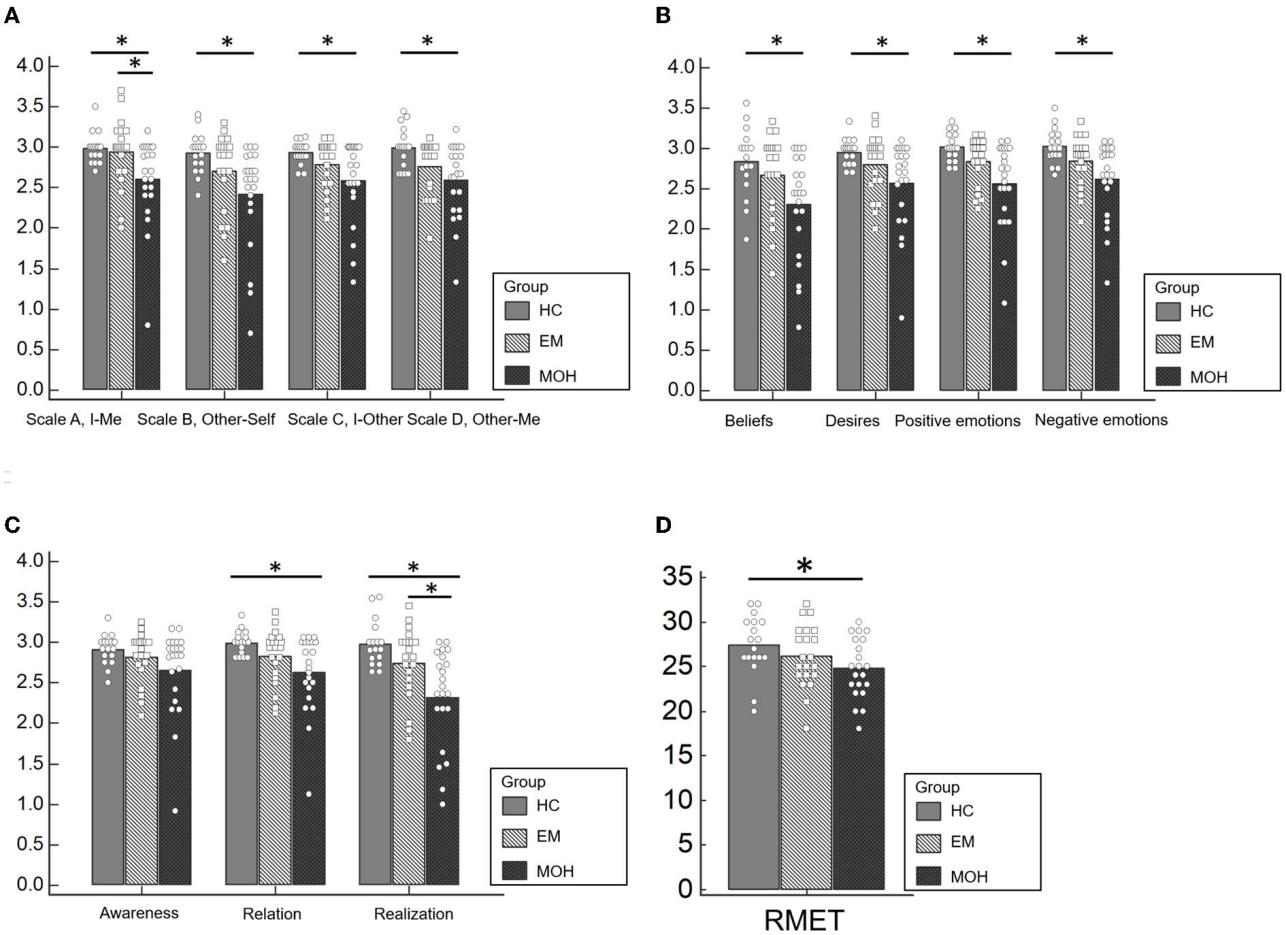
Performances of HC, EM, and MOH groups in ToM battery. **(A)** Represents the results of the ThoMAS in the separate scales. **(B)** Displays the results of the single dimensions of the ThOMAS. **(C)** Shows the results of the subscales of the ThOMAS. **(D)** Displays the results of the RMET. Statistical significant results are marked with ^*^.

In the Scale A, I-Me (Figure 1A), HC performed better (mean value: 2.98 ± 0.19) than EM (mean value: 2.94. ± 0.42); and EM performed better than MOH (mean value: 2.60 ± 0.53) (*p* = 0.009). The *post-hoc* analysis, with a Bonferroni correction applied, showed that the pairwise comparisons were statistically significant comparing HC with MOH (*p* = 0.039) and EM with MOH (*p* = 0.019).

In Scale B, Other-Self (Figure 1A), HC had a better performance (mean value: 2.92 ± 0.25) than EM (mean value: 2.70 ± 0.48) and MOH (mean value: 2.42 ± 0.63) (*p* = 0.004). The *post-hoc* analysis, with a Bonferroni correction applied, showed that the pairwise comparisons were statistically significant comparing HC with MOH (*p* = 0.004).

In Scale C, I-Other (Figure 1A), HC performed better (mean value: 2.94 ± 0.14) than EM (mean value: 2.79 ± 0.30); and EM performed better than MOH (mean value: 2.58 ± 0.50) (*p* = 0.039). The pairwise comparisons were statistically significant comparing HC with MOH (*p* = 0.033).

Similarly, In Scale D, Other-Me (Figure 1A), HC performed better (mean value: 2.99 ± 0.25) than EM (mean value: 2.76 ± 0.34); and EM performed better than MOH (mean value: 2.59 ± 0.46) (*p* = 0.008). The pairwise comparisons were statistically significant comparing HC with MOH (*p* = 0.006).

Regarding the analysis of the single dimensions of the ThOMAS, we found a statistically significant difference between the three subgroups (CT, EM, and MOH) in Beliefs (*p* = 0.015), Desires (*p* = 0.023), Positive Emotions (*p* = 0.004), and Negative Emotions (*p* = 0.008) (Figure 1B).

Concerning the analysis of the subscales, a statistically significant difference emerged between the three subgroups (CT, EM, and MOH) in Relation (*p* = 0.017) and Realization (*p* = 0.001) (Figure 1C). The results of the pairwise comparisons are shown in Table 2.

### Reading the Mind in the Eyes Test (RMET)

A significant difference between groups was detected in the RMET (Figure 1D) (*p* = 0.039). Pairwise comparisons revealed that MOH patients performed worse (mean value: 24.76 ± 3.36) than HC (mean value: 27.39 ± 3.35). No significant difference was observed comparing HC with EM.

### Toronto Alexithymia Scale (TAS-20)

Respectively in the EM group and the MOH group, three patients (19.05%) and seven patients (31.82%) exhibited frank alexithymia. We found a significant difference between the three groups in alexithymia (*p* = 0.023). The *post-hoc* analysis showed higher levels of alexithymia in the MOH group compared to the HC (*p* = 0.033). We also observed a statistical trend in the comparison between EM and HC (*p* = 0.066) (23) (Table 3).

#### Other psychopathological evaluations

#### Hamilton Anxiety Rating Scale (HAM-A)

Experienced anxiety varied significantly between the three groups (*p* = 0.001), with MOH patients reporting higher levels of anxiety than HC on the HAM-A scale (*p* = 0.001). The mean score of the MOH group was 19.77 ± 11.43, which is indicative of moderate anxiety (Table 3).

#### Hamilton Depression Rating Scale (HAM-D)

Although no difference emerged between the groups in terms of depression (HAM-D; *p* = 0.101), the MOH group reported mild to moderate depression (mean score: 14.36 ± 7.74) (Table 3).

#### Dissociative Experiences Scale-II (DES-II)

No significant difference between the three groups was found in dissociative symptoms (*p* = 0.350), with all DES-II mean scores lower than the cutoff for psychopathology (Table 3).

### Correlation between performances in the ToM battery and headache characteristics

Considering the whole clinical group (EM and MOH), a significant correlation was observed between the mean score of Scale A, I-Me, the frequency of headache (MHD: Spearman's rho = −0.398, *p* = 0.008), and the number of drugs per month (Spearman's rho = −0.313, p = 0.041). No significant correlation emerged between MHD and the number of drugs per month and the other ToM scores (ThOMAS and RMET) (*p* > 0.1) (Table 4).

**Table 4:** Correlation between performances in the ToM battery and headache characteristics.

		**Scale A, I-Me**	**Scale B, other-self**	**Scale C, I-other**	**Scale D, other-Me**	**RMET**
Monthly headache days (MHD)	Spearman's *rho*	**−0.398**	−0.216	−0.120	−0.123	−0.160
	*p*-value	**0.008**	0.163	0.445	0.433	0.306
Monthly number of symptomatic drugs	Spearman's *rho*	**−0.313**	−0.175	−0.024	−0.102	−0.089
	*p*-value	**0.041**	0.261	0.879	0.516	0.571

RMET, Reading the Mind in the Eyes; MHD, monthly headache days. Statistical significant results (*p* < 0.05) are shown in bold type.

## Discussion

The main finding of this study is the reduced social cognition skills in patients with MOH, as revealed by poor performances in Reading the Mind in Eyes Test, in all the four subscales of ThOMAS (I-Me, Other-Self, I-Other, and Other-ME), in each of the four types of ToM mental states subscales (Beliefs, Desires, Positive emotions, and Negative emotions), and two out of three dimensions subscales (Relation and Realization).

Theory of Mind is a multidimensional construct that refers to the cognitive ability to understand and predict one's own and other person's mental states, in terms of knowledge, beliefs, intentions, and emotions (8). ToM capacities have been consistently linked to social adaptation and resulted to be affected in a broad range of neurological and psychiatric conditions (24), such as autism spectrum disorders (25).

Our results suggest that MOH patients may have a deficit of awareness and recognition of their own and others' emotions, and it can be hypothesized that this is related to a deficit of social adaptation, limited consciousness, and reduced perception of the somatic and psychological effects of the excessive use of symptomatic drugs.

Furthermore, in our study, patients with MOH showed clear alexithymic traits when compared to healthy controls and episodic migraineurs. These findings are in line with those found in the limited existing literature (26). Alexithymia refers to a specific disturbance in psychic functioning characterized by the difficulty to experience and express one's emotional states and excessive preoccupation with physical symptoms (9). A few studies on alexithymia and primary headaches have been conducted (12), showing that alexithymia may be considered a potential characteristic trait of episodic, chronic migraine, and MOH (10, 26). ToM and alexithymia are different psychological constructs that have in common the recognition of emotions. Moreover, high levels of alexithymia are suggestive of impairment of social cognition. The two concepts might be a continuum of one other (9). Therefore, this relationship existing between ToM and alexithymia is consistent with the altered profile of both ToM and alexithymia in our cohort of patients with MOH.

Regarding other psychopathological findings, patients with MOH demonstrated higher levels of anxiety when compared to HC and EM, while depression was not significantly different in the three subgroups. However, the MOH group reported mild to moderate depression symptoms. Our results confirm the findings from previous studies, investigating the behavioral and psychopathological profile of patients with MOH (1, 27). Regarding the dissociative aspects, in our study, there were no differences among the three groups, even if few studies demonstrated that dissociative symptoms are more frequent in patients with migraine and, in particular, in chronic forms of migraine (28). However, dissociative symptoms in patients with MOH have not been investigated so far.

The overuse of symptomatic drugs in MOH patients might be related to some psychological states, such as fear and anticipatory anxiety of the forthcoming headaches attacks. It can also be hypothesized that anxiety itself may contribute to difficulties in social adaptation. Furthermore, some Authors interpreted MOH as belonging to the spectrum of addictive behaviors (5, 29), and it was associated with obsessive-compulsive tendencies (4). Additionally, this complex psychopathological profile may be linked to chronic drug use in patients with migraine, some of which with possible psychotropic effects, such as opioids and caffeine. Indeed, impairments in social cognition have been extensively studied in patients with other substance use disorders: chronic cannabis, cocaine, alcohol, methamphetamine users, and opioid-dependent patients demonstrated an alteration in some tasks of ToM (30). Mechanisms triggered by recurrent attacks of headache and by drug overuse can concur in the induction of central sensitization observed in MOH. Similarly, drugs of abuse can produce sensitization after a protracted exposure and, intriguingly, substances of abuse and pain seem to activate shared pathways (5).

Studies on functional neuroimaging and connectivity showed that the dorsal medial prefrontal cortex, the temporoparietal junction, and the orbitofrontal cortex represent the anatomical and functional substrate of ToM (6). ToM integrated circuits also engage neural activation of other regions that include the amygdala, the anterior cingulate cortex, and the superior temporal sulcus (31). Moreover, studies have led to the intriguing hypothesis that the dopaminergic and serotonergic systems seem to be engaged in mentalizing abilities (32). The network involved in compulsive drug-seeking is the striate-thalamo-orbitofrontal circuit. The striato-thalamo-orbitofrontal circuit has also been associated with OCD (33). The orbitofrontal cortex plays a critical role in the multiplicity of states and functions, including compulsive behaviors, emotions, and the processing of reward and, its dysfunction may trigger the behaviors that result in compulsive drug administration (34). In patients with abuse of psychotropic substances, the orbitofrontal cortex and anterior cingulate cortex are the most frequently implicated areas in drug addiction, which are both crucial structures for decoding others' emotional mental states (35). In the same way, a persistent orbitofrontal hypofunction has been observed in patients with MOH (36).

Both psychiatric comorbidities and overuse of acute medications are known risk factors for the transformation of episodic into chronic headache (1, 37). In turn, medication-overuse headache seems to be prompted and sustained by psychological disturbances and psychiatric comorbidities. Psychopathological dysfunctions are also possible predictors of relapses and scarce response to treatments (4, 29). Thus, comorbidity between MOH and psychiatric disorders may affect the prognosis of these patients. In addition, MOH patients seem to be characterized by a “neurotic profile” with concerns about physical symptoms and low self-esteem (38). The impairment of different facets of sociocognitive functioning may also be associated with limited insight into illness, interpersonal problems, and social stress leading to increased substance use.

Overall, our findings showed that ToM impairment could be a relevant psychological risk factor for MOH. Treatment strategies for MOH patients are debated, and drug withdrawal remains the gold-standard treatment for this condition (1). Non-pharmacological approaches for the treatment of MOH are equally important. The combination of different forms of psychotherapy, including cognitive behavioral and psychodynamic psychotherapy, and pharmacological therapy may have better chances, more than pharmacological therapy alone, of reducing the burden of migraine and further relapses (39, 40).

## Conclusion

This study may provide a step toward a better understanding of the psychological profile of patients with migraine and medication-overuse headache. Patients with MOH showed a subtle psychopathological pattern characterized by impaired social adaptation. This psychological profile suggests the importance of optimizing the approach toward patients with headache. A careful preliminary evaluation of the psychological profile, including ToM assessment, should be included in routinely patients' evaluation to identify risk factors that may contribute to the chronicization of headache or the maintenance of mechanisms of drug abuse. Finally, we suggest that treatment strategies should include drug withdrawal along with a pharmacological and a non-pharmacological approach.

## Data availability statement

The raw data supporting the conclusions of this article will be made available by the authors, without undue reservation.

## Ethics statement

The studies involving human participants were reviewed and approved by Fondazione Policlinico A. Gemelli IRCCS. The patients/participants provided their written informed consent to participate in this study.

## Author contributions

MR and ST played a lead role in conceptualization, investigation, methodology, project administration and writing of original draft, and data curation. ER and PQ played a role in data curation, methodology, and project administration. MS played a role in conceptualization, methodology, and project administration. CV played a lead role in the conceptualization, formal analysis, methodology, visualization, data curation and writing and editing, and supervision. PC played a lead role in conceptualization, methodology, visualization, writing and editing, and project administration and supervision. All authors contributed to the article and approved the submitted version.

## Conflict of interest

The authors declare that the research was conducted in the absence of any commercial or financial relationships that could be construed as a potential conflict of interest.

## Publisher's note

All claims expressed in this article are solely those of the authors and do not necessarily represent those of their affiliated organizations, or those of the publisher, the editors and the reviewers. Any product that may be evaluated in this article, or claim that may be made by its manufacturer, is not guaranteed or endorsed by the publisher.
